# The elimination of the dengue vector, *Aedes aegypti*, from Brisbane, Australia: The role of surveillance, larval habitat removal and policy

**DOI:** 10.1371/journal.pntd.0005848

**Published:** 2017-08-28

**Authors:** Brendan J. Trewin, Jonathan M. Darbro, Cassie C. Jansen, Nancy A. Schellhorn, Myron P. Zalucki, Tim P. Hurst, Gregor J. Devine

**Affiliations:** 1 QIMR Berghofer Medical Research Institute, Mosquito Control Laboratory, Royal Brisbane and Women's Hospital, Brisbane City, Australia; 2 CSIRO, Agriculture, Dutton Park, Brisbane, Australia; 3 University of Queensland, School of Biological Sciences, St Lucia, Brisbane, Australia; 4 Queensland Health, Metro North Public Health Unit, Herston, Brisbane, Australia; 5 Eliminate Dengue, Institute of Vector-Borne Disease, Monash University Clayton, Melbourne, Australia; Faculty of Science, Mahidol University, THAILAND

## Abstract

*Aedes aegypti* (L.) (Diptera: Culicidae) is a highly invasive mosquito whose global distribution has fluctuated dramatically over the last 100 years. In Australia the distribution of *Ae*. *aegypti* once spanned the eastern seaboard, for 3,000 km north to south. However, during the 1900s this distribution markedly reduced and the mosquito disappeared from its southern range. Numerous hypotheses have been proffered for this retraction, however quantitative evidence of the mechanisms driving the disappearance are lacking. We examine historical records during the period when *Ae*. *aegypti* disappeared from Brisbane, the largest population centre in Queensland, Australia. In particular, we focus on the targeted management of *Ae*. *aegypti* by government authorities, that led to local elimination, something rarely observed in large cities. Numerous factors are likely to be responsible including the removal of larval habitat, especially domestic rainwater tanks, in combination with increased mosquito surveillance and regulatory enforcement. This account of historical events as they pertain to the elimination of *Ae*. *aegypti* from Brisbane, will inform assessments of the risks posed by recent human responses to climate change and the reintroduction of 300,000 rainwater tanks into the State over the past decade.

## Author summary

We examined the historical role that water storage practices and the enforcement of anti-mosquito regulations played in the elimination of *Aedes aegypti* from Brisbane, a major urban centre in Australia. We examined changes in regulations pertaining to mosquitoes, collected government records documenting surveillance, and the response by the community to the actions of local authorities. Our findings indicate that anti-mosquito regulations, underpinned by effective implementation, were successful in gaining community support and removing the risk of mosquito presence at non-compliant properties. In particular, we argue that the removal of rainwater tanks which provided a permanent larval habitat in otherwise suboptimal environments, played a major role in the elimination of the species from Brisbane. Public Health regulations were supported by a large surveillance effort by local government health officers that were empowered to enforce legislation where necessary. Our findings are of importance to health authorities managing the ongoing expansion of *Aedes* populations, particularly in regions of sub-optimal climate and where water storage has become a major concern.

## Introduction

Dengue fever is the 21^st^ Century’s most important mosquito-borne viral illness, exerting a huge economic and health burden in the tropics and sub-tropics [1]. The incidence of dengue has increased 30 fold over the past five decades and the disease is now estimated to affect up to 390 million people each year [1, 2]. This increase in prevalence is due to substantial growth in urbanization, trade, international travel, and the spread of its major vectors [3]. Historically, the high infection rate of the disease has exerted a severe toll on development and economic progress through lost productivity and costs of mosquito control activities [3–5].

In Australia, four large dengue epidemics that swept through Queensland and New South Wales between 1897 and 1926, were responsible for a reported 733 deaths, and affected up to 90% of the urban population in each outbreak [6]. Dengue was first isolated in Australia around 1956, and before this time all dengue cases were based solely on clinical diagnoses [7, 8]. More recently, a rise in imported cases of mosquito borne diseases into Australia has increased the risk of local transmission of dengue, chikungunya and Zika viruses in all regions where the primary vector, *Aedes aegypti* (L.) is present [9, 10].

**Fig 1:**
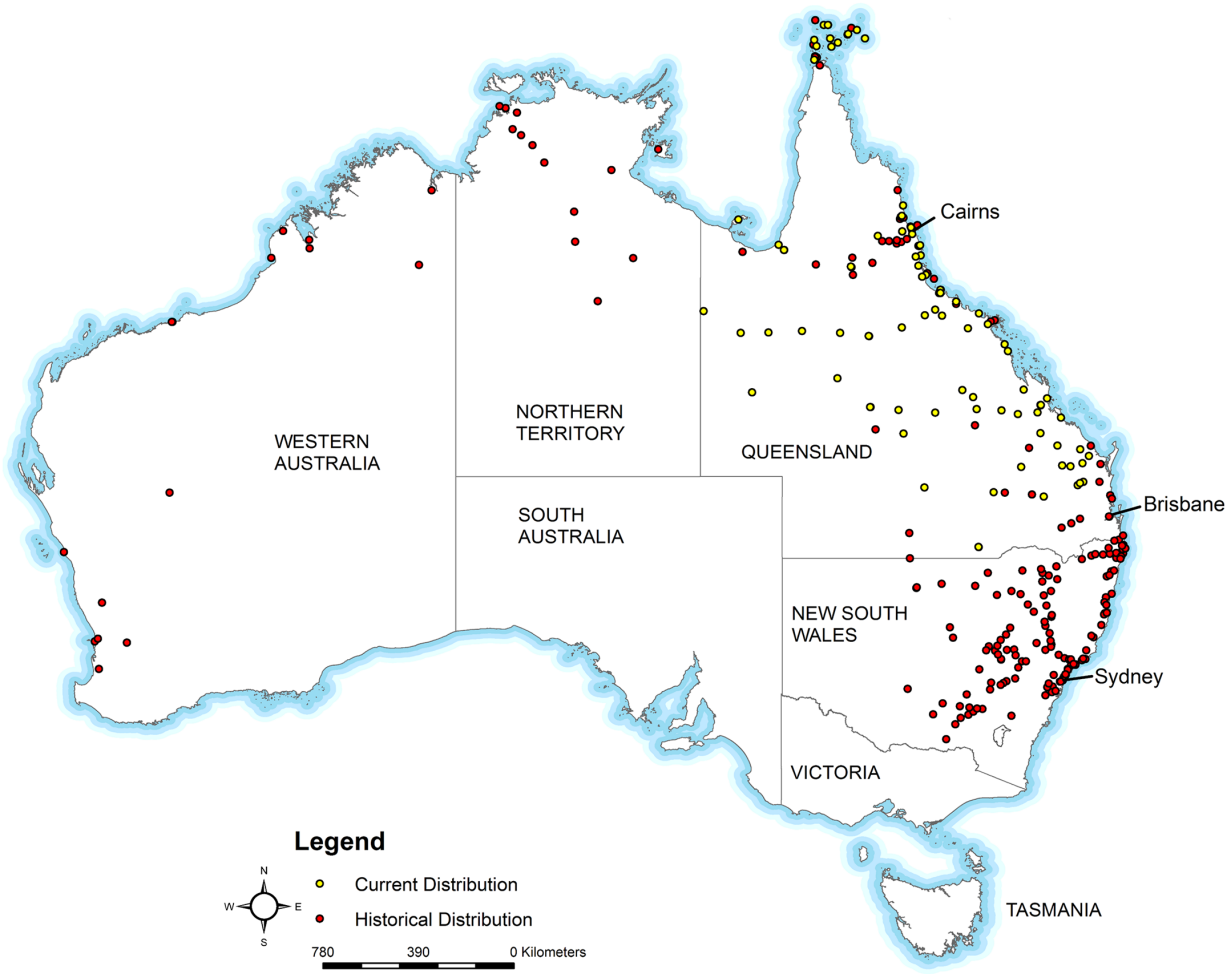
Historical (red dots) and contemporary (yellow dots) distribution records of *Aedes aegypti* in Australia 1887–2016. Location records taken from Beebe et al. [17] and S1 List. Map base layer sourced from Australian ABS digital boundary data [18] and licensed under CC [19].

*Aedes aegypti* is most abundant in northern and some parts of central Queensland. Populations have recently been discovered in the South East of the State, 170 km north of Brisbane, the state capital [11]. It was in Brisbane that Thomas Bancroft first implicated *Ae*. *aegypti* as an agent in dengue transmission in 1906 [12], and this was later confirmed by Cleland in 1917 [13]. At the start of surveys for *Ae*. *aegypti* in 1911, Brisbane occupied ~77 km^2^ (30 mi^2^) and by 1923 had grown to ~163 km^2^ (63 mi^2^)[14]. Brisbane is the largest urban centre of the State with a population of 1.18 million inhabitants [15], an area of 1,338 km^2^ (516 mi^2^) and it lies just outside of the southern margin of *Ae*. *aegypti* in eastern Australia (Fig 1) [16].

*Aedes aegypti* has co-evolved with and feeds predominantly upon humans, and uses the many types of artificial containers found in urban locations for oviposition [20, 21]. As such, this mosquito species is closely associated with the domestic environment and the many microclimates found there [22]. Eggs are desiccation-resistant and can stay dormant during unfavourable periods [21]. Therefore, presence of the species is not entirely dependent on rainfall for the provision of suitable aquatic larval habitats and its persistence is facilitated by the use of artificial water-holding containers [23, 24]. The unscreened house design typical of Queensland in the early 1900s represented highly favourable habitat for *Ae*. *aegypti* adults, providing unrestricted access to a human blood meals and enhancing disease transmission [25]. Once established, *Ae*. *aegypti* disperses readily through the urban environment and suitable habitat is constantly being invaded [26].

In the past, these characteristics have allowed *Ae*. *aegypti* populations to persist throughout much of Australia. Accordingly, its historical distribution includes Queensland, the Northern Territory (NT), Western Australia (WA; including the south) and New South Wales (NSW; Fig 1), with unconfirmed reports in Victoria [27, 28]. This range decreased dramatically in the mid-twentieth century, with the last recorded collections in NSW in 1948, and WA in 1970 [29]. In the NT the last recorded collection was in 1957 [27], but there were some temporary establishments in 2004 and 2011 which were subsequently eliminated [30, 31]. Herein we refer to contemporary term “elimination” as the intentional action by humans to remove *Ae*. *aegypti* from a defined geographical area [32]. Historically, this action has been referred to as “eradication” by medical entomologists [33–37]. We refer to the elimination of dengue as the reduction of disease transmission to zero incidence in a defined geographical area [32]. In Brisbane during the early 1900s, *Ae*. *aegypti* was highly abundant and government agencies set out to eliminate it based on the control successes (that targeted larval habitat) observed during the construction of the Panama Canal [38–44]. At some time after the last outbreak of dengue in 1943, the mosquito was assumed to have disappeared from Brisbane. There are a number of hypotheses suggested by medical entomologists for the wide-spread geographic disappearance of the species, however there are little quantitative data to support these assertions and whether or not they apply to Brisbane [29, 45].

Rainwater tanks have historically been a conspicuous feature of the Australian rural and urban landscape. Since their introduction in the mid-to-late 1800s, these large structures provided a reliable source of potable and non-potable water to households and commercial premises and inadvertently acted as key larval habitat for *Ae*. *aegypti* [27, 29, 43, 46]. In Brisbane, rainwater tanks were first identified as habitat for mosquitoes during the early 1900s [47, 48], and regulations were introduced in 1911 to prevent mosquitoes from exploiting rainwater tanks and dwellings [49]. Permanent water storage containers provide a reliable population source by acting as refugia for mosquitoes during times of sub-optimal climatic periods such as drought or extreme seasonal temperatures [23, 27, 50, 51]. Recent modelling suggests the presence of rainwater tanks was an important factor in the historical distribution of *Ae*. *aegypti* by potentially buffering the effects of climatic extremes on biological processes at geographical range limits [52].

Despite a keen interest in controlling mosquito related diseases in tropical and subtropical countries around the world, there are few documented examples of elimination and little understanding of the factors that have led to localized *Ae*. *aegypti* extinction [53–55]. Here we review data collected from a range of historical sources and evaluate the role of anti-mosquito regulations, surveillance and the removal of rainwater tank infrastructure in the elimination of *Ae*. *aegypti* from Brisbane.

## Methods

We acquired historical records compiled from a range of scientific, government and media sources describing anti-mosquito legislation, mosquito surveillance, regulation enforcement and the presence of dengue and *Ae*. *aegypti* in Brisbane. We then asked a series of questions. Firstly, what evidence is there to test the hypothesis that *Ae*. *aegypti* is still present in Brisbane? Secondly, if local elimination occurred, what quantifiable evidence was there to test the hypotheses that the removal of rainwater tanks contributed to the elimination of the species? Finally, what were the drivers that led to the removal of rainwater tank infrastructure? To answer these questions, we created a time-line of events and linked direct observations recorded by government surveillance and regulatory enforcement.

Scientific publications and annual council and state government reports provide quantitative and subjective measures of a number of factors that potentially affected the historical presence of *Ae*. *aegypti* in Brisbane. We collected data from entomological surveys that estimated the prevalence of rainwater tanks on premises and incidence of mosquitoes within these tanks. From the first surveys in 1911, anti-mosquito policy deemed rainwater tanks non-compliant if they allowed the ingress and egress of mosquitoes [49]. Through enforcement of this legislation, residents found to have larval habitat on their property (including non-compliant rainwater tanks) were warned that they were in violation of mosquito regulations, and given a “notice of breach”. Residents were asked to comply within two months, after which patrol members would return and re-survey the property [56]. If the return survey was again in breach of regulations, the resident could be in default, fined and prosecuted. We only included initial surveys (and excluded re-surveys) in estimations of total dwellings surveyed and for prevalence and compliance rates of rainwater tanks as a reflection of conditions at that time point. Survey results allowed us to identify temporal trends in the outcomes of entomological inspections; prevalence of tanks on properties, rates of rainwater tank compliance with regulations, mosquito presence in tanks, the number of notices given for a breach of mosquito regulations and the number of notices complied with (S1 and S2 Tables). This is the first time those data have been collated to demonstrate the role of government in the elimination of *Ae*. *aegypti* from Brisbane.

All *Ae*. *aegypti* ‘sightings’ were based on scientific or government publications where identification to species level was done by entomologists (S2 List). To test the hypothesis that *Ae*. *aegypti* was still extant in Brisbane over the period from 1887 until 2016, we applied the method of Jaric and Ebenhard [57]. Briefly, this method extends the work of Solow [58] who provided an equation for inferring extinction based on sighting records over time. Here sightings during the observation period are arranged from first to last and used to express the probability of species presence in relation to the number of time units in which the species was recorded [58]. Authors using this method use *P* = 0.05 as the probability threshold, below which the species can be regarded as extinct [47].

Next, we examine the evidence for whether the mosquito population is extinct by first estimating the probability, *p*, that mosquitoes are observed each year (based on 27 sightings over the first 69 years), and secondly, estimating the probability that there would be 59 subsequent years of no sightings given the estimated value of *p*. The probability of observing mosquitoes from one year to the next, *p*, was estimated as $\hat{p}$ using maximum-likelihood, and a 99% confidence interval, [*p*_*l*_,*p*_*u*_], was created by inverting a likelihood ratio test-statistic. The confidence interval includes all the values of *p* for which

$$l\left(p;x\right)\geq l\left(\hat{p};x\right)-3.317,$$

where *l*(*p*;*x*), is the log-likelihood function evaluated with a yearly sighting probability of p, x denotes the observed historical data, and 3.317 is half the 99th percentile of a *χ*^2^ random variable with one degree of freedom.

Finally, we use the optimal linear estimation (OLE) function from the R package sExtinct() to estimate the date of extinction, with 95% confidence intervals. The OLE method infers time to extinction from a temporal distribution of species sightings [59]. All models and simulation code were created and run in R v3.3.2 [60].

We estimated rates of dengue during historical outbreaks in Brisbane using minimum infection rates documented in the literature during epidemics (70%), and population data obtained from government statistics [6, 13, 61–63]. Deaths associated with dengue during epidemics were taken from historical medical literature and government population statistics [6, 13, 61]. As case data were not technically confirmed, it is possible that some were outbreaks may have been undiagnosed cases of chikungunya or other viral diseases [6, 64, 65].

Brisbane was first declared a city in 1902 and by 1911 contained 26,645 private dwellings [66]. In 1925, the city amalgamated with a number of other local authorities to become Greater Brisbane and expanded from 43,935 dwellings in 1921 to 68,096 by 1933 [67, 68]. We used government statistics describing the number of dwellings within the council boundary of Brisbane annually from 1895 until 1971 to estimate the total number of tanks (proportion of dwellings in surveys that had a tank present multiplied by total dwellings in Brisbane for relevant year), total non-compliant tanks (proportion of non-compliant tanks surveyed multiplied by total estimated number of tanks), the number of non-compliant tanks with mosquitoes present (estimated non-compliant tanks multiplied by proportion of tanks with mosquitoes present in surveys) and the proportion of non-compliant tanks to total dwellings (estimated total non-compliant tanks divided by total dwellings)[61].

## Results

### What evidence is there for the presence of *Aedes aegypti* in Brisbane?

Noting that historic records refer to *Ae*. *aegypti* by multiple names (*Culex bancroftii*, *Stegomyia fasciata*, *Aedes argenteus* and *Stegomyia calopus*; see [69]), the first published record of *Ae*. *aegypti* in Brisbane was in 1887 [70] and we recorded a total of thirty-seven references from historical records (Table 1, S2 List). Entomological, house-to-house surveys for the species did not commence until anti-mosquito regulations were introduced in 1911 and, at this time, *Ae*. *aegypti* was highly prevalent across the city [14, 71].

**Table 1:** Estimated locally acquired dengue cases based on a 70% incidence of dengue, recorded deaths due to dengue and number of references to the presence of *Aedes aegypti* in Brisbane from historical records.

Decade	Dengue cases*	Deaths^#^	Reference to *Ae*. *aegypti*^†^
1890–1899	77,200	40	1
1900–1909	87,970	108	4
1910–1919	Not recorded	62	12
1920–1929	184,597	66	8
1930–1939	2	0	4
1940–1949	664	0	7
1950–1959	0	0	1
1960–1969	0	0	0
1970–1979	0	0	0
**Total**	**350,438**	**276**	**37**

* Number of dengue cases estimated from a minimum of 70% incidence rate within the Brisbane metropolitan area. Incidence rate in the literature was estimated between 70–90% in historical medical records during large outbreaks [6, 13, 61–63].

^#^ Deaths recorded from scientific literature and government vital statistics [13, 61].

^†^ References to *Aedes aegypti* in Brisbane from scientific and government reports [S2 List]. The reader should note that *Aedes aegypti* was first implicated as a vector of dengue in 1906 [12] and confirmed by 1917 [13].

By 1932, mosquito control was decentralized to local health inspectors and little species-specific data was recorded in house-to-house surveys [56]. A dengue outbreak during 1943 re-intensified mosquito surveys with identification to species (Table 1)[72]. However, once the Second World War was over the role of mosquito surveillance was, again, taken over by generalist local government officers, and council reports after 1947 no longer identified or recorded mosquito presence to species level classification. Subsequent recordings of *Ae*. *aegypti* were made by Elizabeth Marks who documented distributions from 1957 until 1981, surveying the last accounts of larval *Ae*. *aegypti* in Brisbane in 1944 [73], 1948 [74] and 1957 (Table 1)[75].

When inferring extinction likelihood, the total length of the observation period (T) was 128 years (1887 until 2016), the total number of years where *Ae*. *aegypti* was observed (n = 27), and the period between the last positive survey and last observation was 59 years (1957 until 2016). Results suggest the probability that the species is still present based on the average frequency of sightings was calculated as *P* = 0.042. This value was below our threshold level (*P* = 0.05) and suggests the population does not exist. Using the maximum-likelihood and inverted ratio test-statistic approach, we estimated $\hat{p}=0.391$ and assuming the true value of $\hat{p}$ is in the 99% range, we estimate [*p*_*l*_,*p*_*u*_] = [0.257,0.546]. The probability of observing 59 consecutive years with no sightings were estimated from the probability mass function of the binomial distribution as 2.45E-8 and 5.83E-21 respectively. Therefore, based on the inferred sighting probabilities, there is very little evidence to suggest that the mosquito population is extant given the 59 consecutive years of absence. Finally, using OLE we estimate that from 37 total observations in Brisbane, the species likely went extinct in 1961 (upper CI = 1974, lower CI = 1957).

### Historical records of dengue in Brisbane

A series of dengue outbreaks in Brisbane during the late 19^th^ and early 20^th^ century took a severe toll on the population of Brisbane and its economy [6]. Deaths due to dengue peaked in 1905 and infection rates of 70–90% were reported during all epidemics across the city and until 1926 (Table 1)[13, 63, 76, 77]. The last outbreak in Brisbane during 1942/43 resulted in 646 cases [72] and was precipitated by epidemics occurring in northern Queensland [78]. The last recorded, locally acquired case of dengue in Brisbane was noted in 1948 [74].

### Rainwater tanks as potential larval mosquito habitat

**Fig 2:**
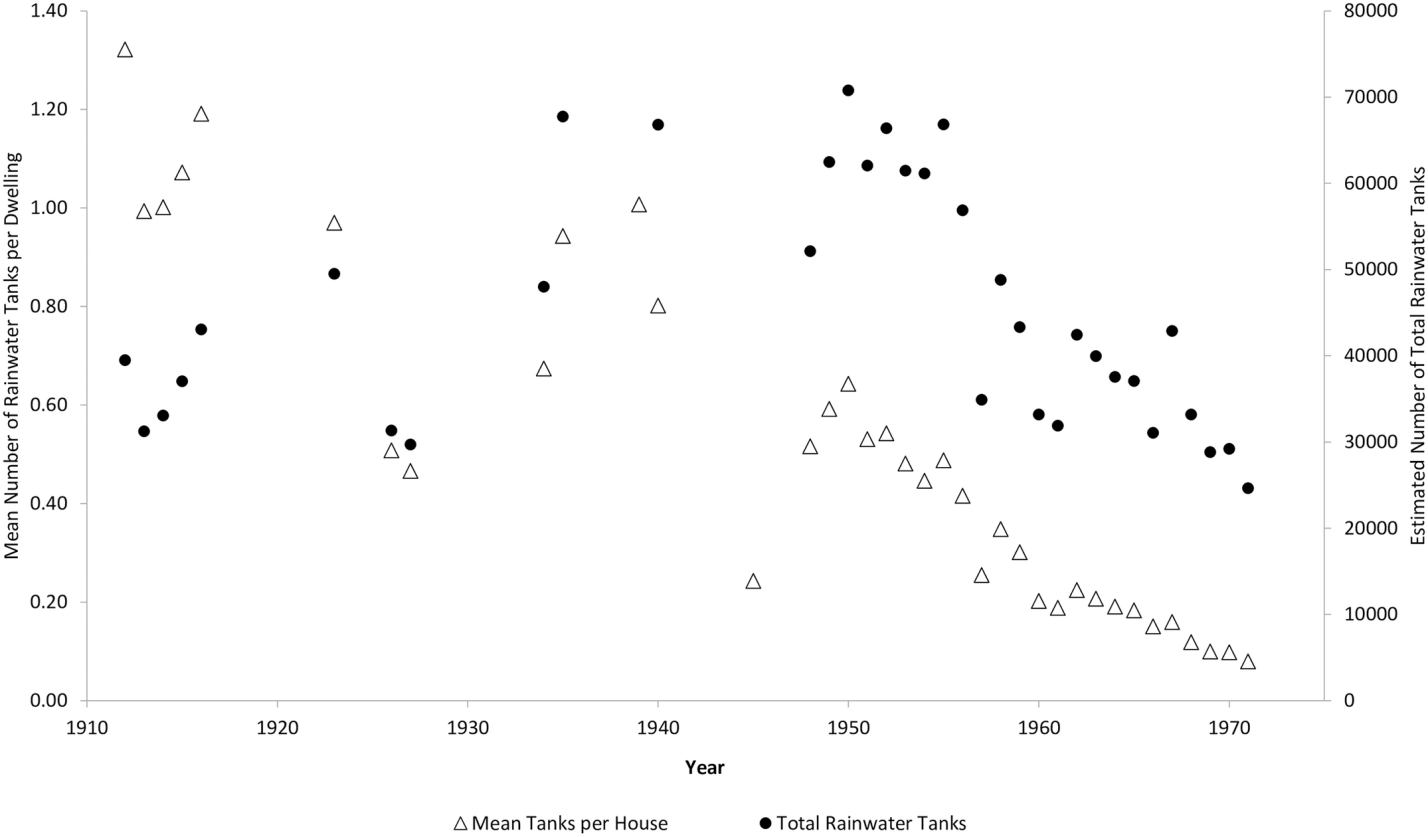
Estimated mean number of rainwater tanks per dwelling (primary axis) and estimated total number of domestic rainwater tanks (secondary axis) per year in Brisbane, 1912–1971.

Shortages in reticulated water in Brisbane during the 1800s and early 1900s meant rainwater tanks provided the most reliable source of potable drinking water for use around the home [79]. The mean number of water tanks declined from over 1 per dwelling in 1912 to less than 1 in 10 in 1971 (Fig 2). House-to-house surveys were conducted systematically after the creation of the Brisbane Entomological Section in 1926, with the city divided up into 88 blocks and house-to-house patrols responsible for surveying a number of blocks per year [42]. During the severe dengue epidemic years from 1897 to 1927, the mean number of rainwater tanks per dwelling were at the highest recorded (Fig 2; mean ± *SD* = 0.94 ± 0.3). Following the introduction of a large water reservoir to supply Brisbane in 1954, the mean number of rainwater tanks per dwelling consistently fell below 0.50 tanks. Based on the number of dwellings within the city area, we estimated the total number of rainwater tanks at 39,513 (95% *CI* ± 0) in 1912, before reaching a maximum of 70,794 (95% *CI* ± 515) in 1950, and declining to 24,647 (95% *CI* ± 935) by 1971 (Fig 2).

### The role of regulations and compliance on the prevalence of mosquito habitat

**Fig 3:**
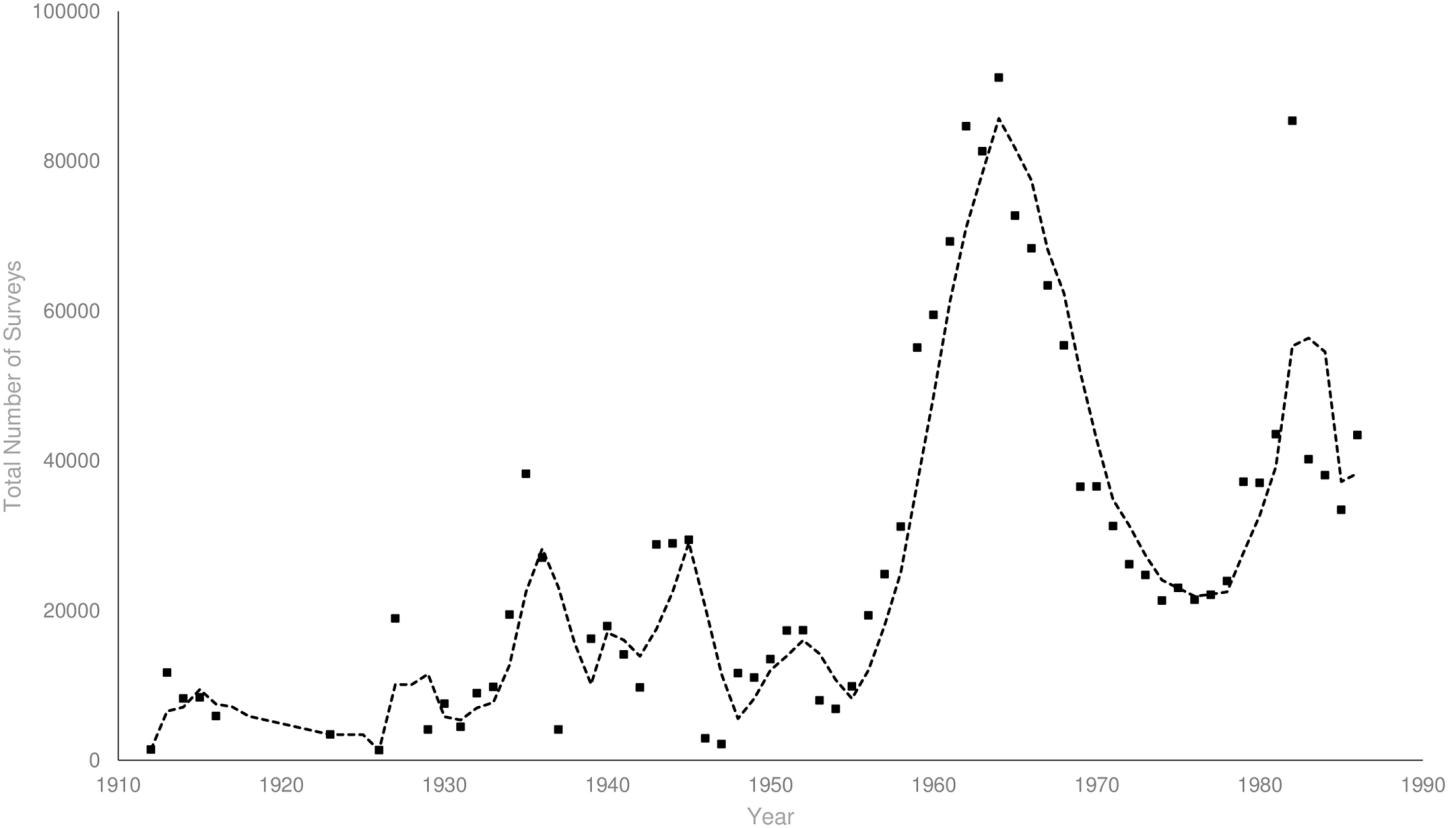
Dwellings surveyed for breaches in mosquito regulations from 1912 until 1989 in Brisbane, Australia. Trend line represents a three year moving average.

The total number of house-to-house surveys to monitor compliance and prevalence of mosquitoes per year by entomologists and inspectors has fluctuated greatly (Fig 3). The period after the Second World War is of most interest, because this was when the City Council focused the most effort on the elimination of *Ae*. *aegypti* [80]. During this period, there was a large increase in annual house-to-house surveys, from 11,158 in 1948, to 91,127 in 1964. The number of surveys decreased after 1964 with the mean dropping to 37,436 (95% *CI* ± 8,041) per year until 1989. Surveys no longer recorded entomological data after 1971, although inspectors continued to give notices of breaches in regulations until surveys were discontinued in 1989.

**Fig 4:**
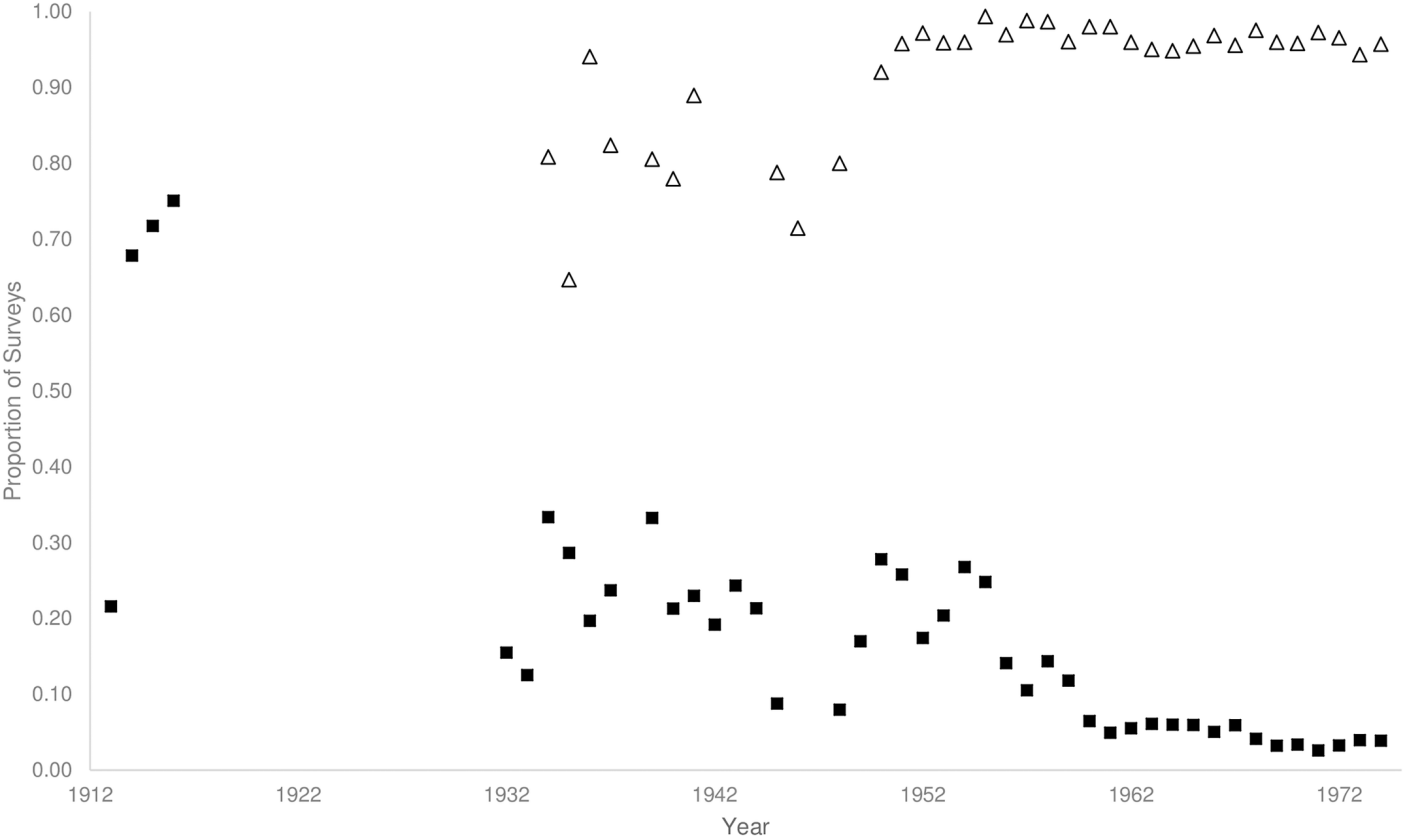
Proportion of surveyed houses resulting in a notice of breach in regulations (squares), and the subsequent proportion of those notices that resulted in compliance (triangles) from 1912 until 1974.

The original anti-mosquito regulations defined mosquitoes as noxious, defined what constituted a breach in regulations, and prescribed ways that the resident must prevent mosquito presence [49]. City Ordinances introduced in 1921 [81] and amended in 1933 [82], were implemented to allow the enforcement of regulations by local authorities and to set penalties for a breach in these regulations (for examples of breaches see Table 2)[83]. The system of notices provided data on breaches with regulations “notices complied with”, and provided measurement of the success of surveillance programs. Compliance with anti-mosquito regulations typically involved the resident sealing or removing rainwater tanks that were at risk of breeding mosquitoes and removing or treating other potential containers. Between 1913 and 1954 many surveyed dwellings were in breach of regulations (Fig 4; squares; mean ± *SD* = 0.27 ± 0.18). After that time however, the proportion of surveys resulting in a notice for a breach in regulations dropped (Fig 4; squares; 0.05 ± 0.05), and reached 0.03 by 1974. Compliance with notices was relatively low between 1934 and the end of the Second World War (Fig 4; triangles; 0.80 ± 0.09) but during the 1950s and 1960s (the period when *Ae*. *aegypti* disappeared) most notices resulted in compliance (Fig 4; triangles; 0.95 ± 0.04).

**Table 2:** Key changes in local government legislation to prevent container inhabiting mosquitoes in residential dwellings, Brisbane from 1911 to 1942.

**1911 –**The Health Acts Noxious Vermin (Mosquitoes) order of Governor in Council, Metropolitan Area of Brisbane^1^	**1921 –**Mosquito Prevention and Destruction Regulations, Brisbane^2^	**1933 –**Amendments to Ordinances Greater Brisbane^3^	**1942—**Mosquito Prevention and Destruction Regulations, Brisbane^4^
Declares mosquitoes to be noxious	Prescribes that Local Authorities will be given power to make house-to-house visits to ensure regulations are being complied with, as required by the Director General	Declares type of mosquito habitat that the *resident* is responsible for managing	Declares penalties for breaches in regulations
Prescribes that every tank, cistern or similar receptacle used for the storage of water will be kept protected against the ingress and egress of adult mosquitoes through the use of mesh at every opening (18 meshes to the inch)	Prescribes that a penalty of up to 50 pounds may be given to owners or occupiers interfering with, obstructing, damaging or destroying gutters or drains.	Declares precautionary measures *residents* must apply to mosquito container habitats	Declares officers of Local Authorities to be authorized to act upon the regulations.
Prescribes the use of: kerosene, fish, wire netting or emptying any container (dried and cleaned) every seven days.	Prescribes a penalty for owners or occupiers causing and failing to fill in excavations.	Prescribes the duty of all *residents* to apply the appropriate precautionary measures in respect of all mosquito container habitats: prescribes penalty for default	Prescribes a penalty for a breach in regulations (those mentioned here) of up to 20 pounds, besides cost and expenses for proceedings.
Declares Owners or occupiers must not allow rubbish/containers that could serve as habitat for juvenile mosquitoes		Prescribes the giving of notice of default only in cases in which the resident may reasonably be presumed not to be aware of the default	Prescribes a penalty for failure to comply with a breach in regulations to be no more than 20 pounds.
Declares Owners or occupiers must repair drainage to prevent holding water and habitat for juvenile mosquitoes		Prescribes that in the obstinate default of *residents*, Ordinances may be carried out by the Local Authority at the expense of the resident involved	Prescribes every tank, cistern or similar receptacle used for the storage of water to be kept protected against the ingress and egress of adult mosquitoes through the use of mesh at every opening (16 meshes to the inch, with 28 gauge wire)
Declares Owners or occupiers must use all available means to prevent the presence of mosquitoes on their properties		Prescribes the Chief Inspector shall administer the oversight of the regulations	Prescribes the resident or owner to cut down overhanging vegetation that would deposit leaves in gutters or on tank-tops, at the request of an Inspector
			Prescribes a report to be made of all inspections and forwarded to the Director General.

^1^ [38].

^2^ [84].

^3^ [85].

^4^ [86].

### Non-compliant rainwater tanks as larval mosquito habitat

**Fig 5:**
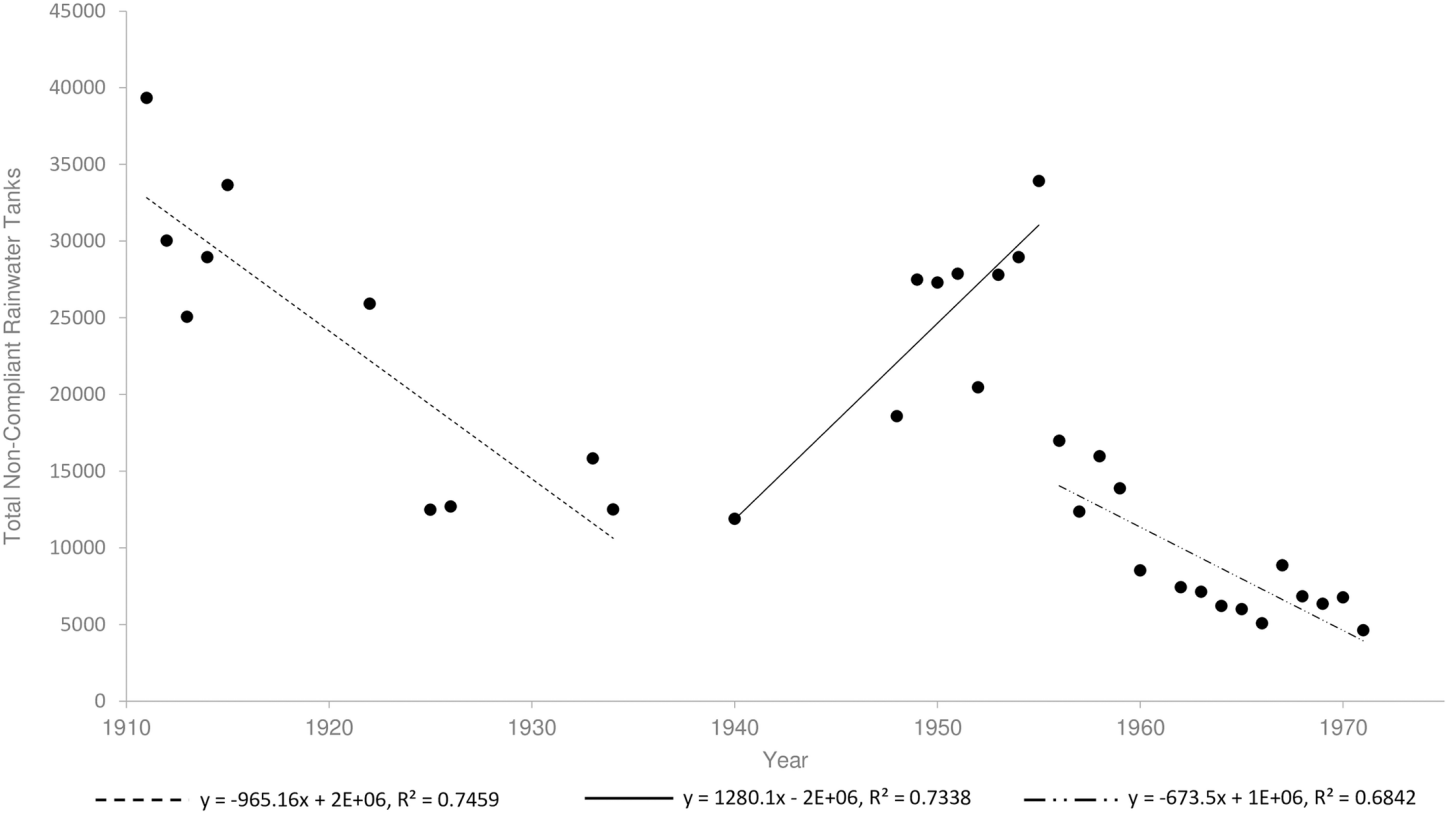
Estimated total number of non-compliant rainwater tanks in Brisbane from 1912 until 1971. Regression lines represent trends in tank compliance during three time periods. Of interest is the second around the Second World War when materials were unavailable for tank maintenance and number of surveys was low (see Fig 3).

Reports by government entomologists provided the first qualified evidence that *Ae*. *aegypti* was present in rainwater tanks from 1912 [71, 87]. Surveys in 1923 and 1927 provide further evidence for the presence of *Ae*. *aegypti* in rainwater tanks [14, 43]. Using survey data, we estimated the total number of non-compliant tanks in Brisbane (Fig 5). When regulations were first introduced in 1911, the majority of tanks were non-compliant [71] and we estimate a total of 39,341 (95% *CI* ± 182) non-compliant rainwater tanks in Brisbane at this time (Fig 5). This was followed by a period of steady decline to 1940 when we estimate 11,900 (95% *CI* ± 293) non-compliant tanks were present (Fig 5). A marked increase in the estimated number of non-compliant tanks was observed after the Second World War (with a maximum of 33,931; 95% *CI* ± 788), due to shortages in labour and materials for repairs (Fig 5)[88]. A rapid decline in the estimated number of non-compliant tanks after 1955 returned to the trends observed before the war, and by 1971 there were only an estimated 4,627 (95% *CI* ± 324) non-compliant tanks left in Brisbane (Fig 5).

### Presence of mosquitoes in non-compliant rainwater tanks

**Fig 6:**
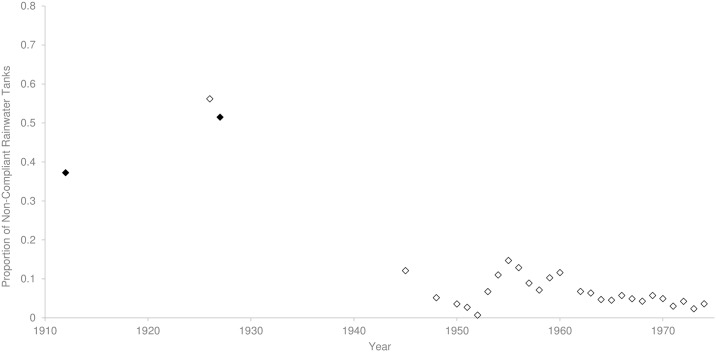
The proportion of non-compliant rainwater tanks surveyed with *Aedes aegypti* (black diamonds) and mosquito presence only (white diamonds) from 1912 until 1974.

Early entomological surveys recorded the number of non-compliant rainwater tanks that contained *Ae*. *aegypti* [71]. The highest proportion containing this species was observed during years when dengue epidemics were most severe (0.37: 679/1,832, *SE* ± 0.011; Fig 6). After the large dengue epidemic in 1925/26, which was associated with 66 deaths, the proportion of non-compliant tanks with *Ae*. *aegypti* was highest at 0.51 in 1927 (1,940/3,768, *SE* ± 0.008; Fig 6)[43]. From that point, the proportion of non-compliant rainwater tanks containing all mosquito species decreased to 0.12 in 1945 (284/2,344, *SE* ± 0.007) and 0.07 in 1952 (20/2,902, *SE* ± 0.002; Fig 6), followed by a further rise to 0.15 in 1955 (359/2,443, *SE* ± 0.007; Fig 6). That increase was probably due to increases in the total number of non-compliant rainwater tanks present during the same period (Fig 5). As surveys did not identify to species at this time, we cannot determine these were *Ae*. *aegypti* or the native container inhabiting species, *Ae*. *notoscriptus*. After 1955, the proportion of infested tanks decreased until the end of entomological surveys in 1971 where container inhabiting mosquitoes were no longer in high prevalence (Fig 6) and non-compliant rainwater tanks were not a common feature in Brisbane’s urban landscape (Fig 5).

**Fig 7:**
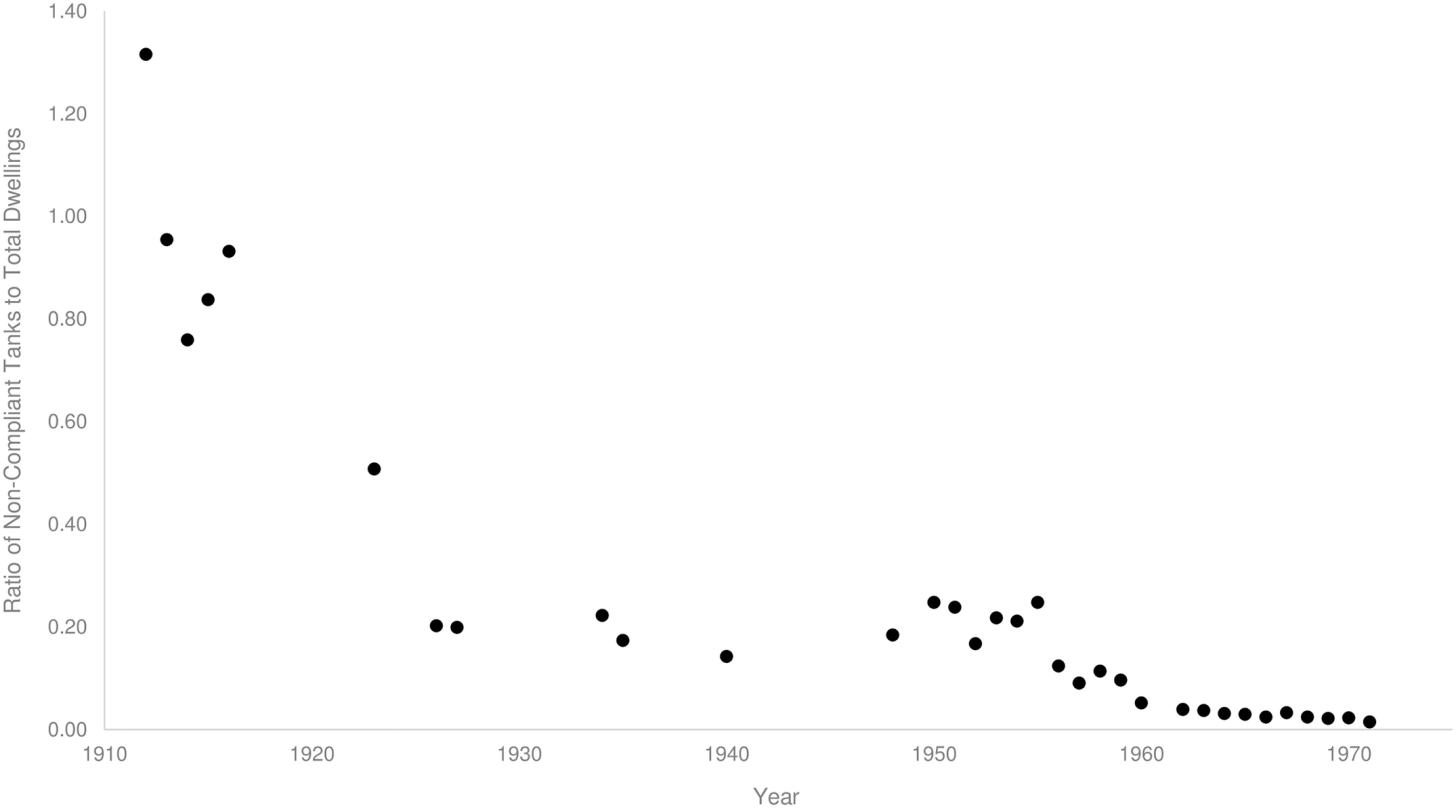
Ratio of the total number of non-compliant tanks to the total dwellings in Brisbane from 1912–1971.

To estimate the density of larval habitat provided by domestic rainwater tanks, we calculated the ratio of non-compliant tanks to dwellings in Brisbane from 1912 until 1971 (Fig 7). High ratios of non-compliant tanks per dwelling (between 1.32 to 0.83 during 1912 and 1923 respectively) relate to periods where epidemic dengue occurred throughout the city. Although the density of non-compliant tanks decreased to 0.2 by 1925/26, the large dengue epidemic during these years may have been facilitated by the high proportion of non-compliant rainwater tanks with *Ae*. *aegypti* present (Fig 6). From 1926, the ratio of non-compliant tanks to dwellings remained constant, at around 0.2 non-compliant tanks per dwelling until 1955. As Brisbane continued to grow and the number of rainwater tanks started to decrease, the ratio of non-compliant tanks per dwelling decreased to 0.08 by 1971 (Fig 7).

## Discussion

### Evidence that *Aedes aegypti* was eliminated and remains absent

The local extinction of invasive species and the decision on whether an elimination program can be declared successful is challenging. Techniques for detecting invasive mosquitoes are imperfect, and failure to detect a species does not confirm absence with certainty. In Brisbane, despite a constant mosquito management and surveillance program, there have been no recorded specimens of *Ae*. *aegypti* outside of first ports of entry or quarantined facilities since 1957. Surveys conducted by Elizabeth Marks (Brisbane) and local health authorities from 1965 until 1980 did not detect the species, and larval surveys in 1995 until 1997 and 2007/08 also failed to isolate the vector in high risk residential areas in Brisbane [69, 73, 89–91]. An extensive larval survey of 4,983 premises from 2010 until 2012 likewise did not detect *Ae*. *aegypti* [92]. Contemporary surveillance efforts have not detected *Ae*. *aegypti* in the Brisbane City Council region to date, though the species has been intercepted during routine surveillance at first ports (Cassie Jansen, Queensland Health, pers. comm 20 May 2016).

The absence of locally-acquired dengue cases in Brisbane since 1948, despite being a notifiable disease, suggests that any population of *Ae*. *aegypti* if established, is no longer sufficient in distribution and abundance to vector the virus. The large decrease in dengue fever cases from the 1926 epidemic when compared to the outbreak in 1943 (Table 1) indicates that the mosquito population had likely been reduced substantially prior to the outbreak. As such, the limited cases observed during the 1943 outbreak suggest there was little opportunity for local transmission (Table 1). Following the large outbreak in Townsville in 1955 (approximately 15,000 cases), no cases of locally acquired dengue were recorded in Brisbane, as noted by Doherty [93] who stated that “Brisbane remained unaffected, although each of the seven previous outbreaks of dengue in North Queensland since 1895 had been followed by a high incidence in Brisbane” and from a council annual report, “University collectors have tried in vain to obtain specimens of the vector (*Ae*. *aegypti*). This position is a direct result on the active eradication policy of the Council” [94]. These suggest that by 1955, *Ae*. *aegypti* was no longer in numbers large enough to vector the virus amongst the population of Brisbane. Between 1948 and the present day (despite high numbers of imported cases in recent years [95]) there have been no locally-acquired cases of dengue fever, suggesting dengue has been eliminated from Brisbane.

Our calculations (based on frequency of sighting records) suggest that *Ae*. *aegypti* is unlikely to be established in Brisbane and it likely went extinct around 1960. Results from the Jaric and Ebenhard [57] method were not highly significant, suggesting there is a small probability of presence. However our maximum likelihood method, where the probability of detection after the last survey date is the same as before it, suggests it is highly unlikely that the species is present after a period of 59 years. We propose, therefore, that *Ae*. *aegypti* is currently either absent from the city or below our ability to detect the mosquito. As *Ae*. *aegypti* continues to exist just north of the region (Fig 1), there is therefore a high risk of future re-establishment. Discontinuing ongoing surveillance could lead to increased future costs in mosquito management and higher disease risk.

### Factors driving the elimination of *Aedes aegypti* in Brisbane

Historically it is likely *Ae*. *aegypti* relied on particular larval habitat features to persist and re-establish through periods of low rainfall [27, 52]. We believe that three major factors contributed to the disappearance of *Ae*. *aegypti* from Brisbane soon after the 1940s.

### Replacement of rainwater tanks

The removal of large domestic water storage containers (rainwater tanks) and their replacement with reticulated water has been suggested as the principle reason for the elimination of *Ae*. *aegypti* in the city [29, 45]. O'Gower [27] suggested the species might be eliminated in areas of low rainfall by ‘a continuous mosquito control program, and complete replacement of rainwater tanks by a reticulated water system’. It is likely that a similar process led to the species disappearance in the Mediterranean region, where commonly-used basement cisterns were gradually replaced with reticulated water [96]. Although Brisbane had reticulated water from the creation of its first dam in 1866, the crude reservoir systems had no purification and the reservoirs had limited capacity and were prone to the effects of drought. Rainwater tanks were relied upon as a dependable, clean source of drinking water. The first purification system was created in 1919 but tank use continued to increase into the 1950s [79]. It was not until the creation of Somerset Dam in 1954 that a reliable, unrestricted and clean water supply was available to the whole city. This coincided with the period when *Ae*. *aegypti* disappeared from the record. The gradual introduction of a clean, reliable source of reticulated water led to the disuse and removal of rainwater tanks throughout the city. Although the removal of these large water storage containers may be considered a tipping point, it was the removal of non-compliant rainwater tanks acting as permanent larval habitat that played an important role in the process of elimination.

### Removal of non-compliant rainwater tanks

The specific decline in non-compliant rainwater tanks from the early 1930s appears to coincide with a decline in *Ae*. *aegypti* presence and a reduction in dengue transmission. Non-compliant rainwater tanks can provide essential refugia for eggs and larvae and act as population sources after unfavourable climatic periods (such as droughts, dry seasons and winter)[27]. It is possible that non-compliant rainwater tanks reflected the general disordered nature of backyards in the early 1920s. The removal of large water storage containers and other larval habitat as a result of house surveys and regulation would have reduced opportunities for population growth and persistence. During peak dengue transmission in the 1920s, ~50% of non-compliant rainwater tanks contained *Ae*. *aegypti*. By the early 1930’s, Hamlyn-Harris [97] observed that “never in the history of the mosquito campaign had there been fewer domestic mosquitoes” in the city, although he did not directly record the presence of *Ae*. *aegypti*. This is also the period where the ratio of non-compliant tanks to dwellings remained constantly low. This general decrease in non-compliant tanks from 1912 until 1940 may have led to the reduction in container-inhabiting mosquitoes (Figs 5 and 7), and to the lower incidence of dengue in the 1943 outbreak. During and after the War, a shortage of materials contributed to another rise in non-compliant tanks from 1940 until 1955 which was combated with increased surveillance and enforcement [88]. The deployment of experienced health inspectors after the War [28] and the considerable number of dwellings surveyed from 1948 until 1964 (Fig 3), was responsible for the subsequent drop in non-compliant tanks. By this time the proportion of non-compliant rainwater tanks containing mosquitoes had decreased substantially when compared to earlier in the century (Fig 6). By 1952 health authorities understood that *Ae*. *aegypti* had been “effectively reduced in numbers to the point where it was no longer a capable link in the chain of [dengue] transmission” [98]. However the elimination campaign continued to “be vigorously waged” [99] in 1964, and by 1966 the species had been “virtually eliminated” [100]. During 1964, half the dwellings in the city were surveyed as part of the effort by government officials to eliminate *Ae*. *aegypti*. By 1971, the proportion of non-compliant rainwater tanks compared to total dwellings in Brisbane was minimal.

#### Alternate larval habitat

Starting in 1912, teams applied a variety of larval suppression techniques to target other species in swamps, sewers, cemeteries and gully-traps [71, 101, 102]. It is likely that the intensive mosquito program run in parallel to house-to-house surveys (see DDT section below) targeted much of that habitat. The objective of the house-to-house surveys was to “eliminate all mosquito breeding places” [56] which included containers such as rubbish, water barrels, jugs and flower-pot saucers [71]. By the time *Ae*. *aegypti* had disappeared from Brisbane, remaining oviposition sites may have been too transient or too rare to allow the species to maintain an established population, particularly in the pre-plastics era.

### Regulations and community engagement

The elimination of *Ae*. *aegypti* over large geographic areas is extremely rare. Successful large scale campaigns have focused primarily on top-down approaches, reliant on international interventions and the use of chemical control [33]. The majority of these elimination campaigns were abandoned due to their unsustainable cost, the development of resistance in mosquito populations and the move to less regimented campaigns [55, 103]. Recently, larval source reduction has been used successfully to eliminate both dengue [104] and *Ae*. *aegypti* from villages in Vietnam [36]. This method focused on a very local, bottom up approach that engaged local communities to take ownership of the problem by targeting water storage (like large cement tanks, wells and ceramic jars) with a biological control organism, *Mesocyclops* [36]. This method provided affordable tools for villages to maintain control for long periods of time but unfortunately has not proven applicable to many other environments and has not been widely adopted [36, 104].

From the inception of anti-mosquito regulations in Brisbane, it was the role of the resident to ensure that their property was not producing mosquitoes. High proportions of breaches in regulations during the period from 1913 until 1955 indicates the initial poor condition of the rainwater tanks in use at that time, and the considerable effort required to reduce the threat they posed. Early in this period (1912–1933), surveys for mosquitoes in and around rainwater tanks were performed by trained public health inspectors and entomologists representing state and local government [43, 71, 14]. The officials that developed the first mosquito control program were under-resourced and were unable to enforce regulations in the newly amalgamated and growing city [56]. With the formation of the Brisbane City Council mosquito control section, the role of surveillance was undertaken by District Health Inspectors who, by 1942, were authorized to enforce regulations [56]. The ability to compel householders to rectify breaches in regulations was a necessary response to a very real public health threat. Compliance with anti-mosquito regulations was variable before and during the Second World War, but by 1955 had reached almost 100%.

When scaled up to the size of a modern city, a top-down elimination approach is doomed to fail if all stakeholders are not engaged and integrated into the management approach. The public plays a crucial role in mosquito control and elimination campaigns. In 1927, Hamlyn-Harris stated, “No mosquito campaign which does not include the question of publicity can ever hope to be successful, and this together with the education of the public has become a very important part of daily activities” and “It’s (*Ae*. *aegypti*) control is only possible provided an educated and sympathetic public cooperate” [43]. We infer that it was the removal of non-compliant rainwater tanks as key larval habitat through the continued enforcement of regulations that played a substantial role in the disappearance of *Ae*. *aegypti* from Brisbane.

### Potential alternative mechanisms influencing elimination

It is likely that alternative mechanisms contributed to the elimination process, however there is little quantitative evidence to show their effect.

#### Improvements to living standards

Broad social trends and improved living standards after the Second World War improved conditions within the domestic dwelling and outdoor yard areas. O'Gower [27] stated that in the early 20^th^ century ‘the yard was depicted as a service yard complementary to the house in which the pseudo-suburban farmer generated resources for living’. These conditions were a reflection on the social and economic circumstances of the early 1900s which saw two depressions (1890 and 1930) and two World Wars [105]. By the 1940s, improving living standards and disposable personal wealth made the traditional uses of the backyard obsolete. Improvements included sewerage systems to remove waste, wash houses moved inside, the popularity of the ‘Hills Hoist’ (outdoor clothes line), the efficient and mass production of animal farming systems, the motorized lawn mower for keeping lawns tidy, domestic appliances such as the refrigerator replaced food cabinets and, the introduction of reliable reticulated water to urban areas that effectively replaced rainwater tanks. The yard or ‘outdoor living area’ had become a space for recreation; an area that was ordered, designed and purposeful [105]. By the 1970s, society had unwittingly removed the majority of larval habitat from around the domestic dwelling. As such, the removal of habitat through the improvement of living standards is likely to have contributed to the elimination of *Ae*. *aegypti* in Brisbane.

#### Use of residual chemicals

Dichlorodiphenyltrichloroethane (DDT) may have contributed indirectly to the elimination of *Ae*. *aegypti* from Brisbane, however government records lack data on the use of the chemical during house-to-house patrols. DDT was first used by the Brisbane Mosquito Control Section in tests on mosquitoes in September 1946 [106]. At the time, surveys for Anopheline mosquitoes were being undertaken after local transmission of malaria occurred, and these mosquitoes were the primary target [107]. After an initial trial period, DDT was approved for use in swamps and was used to target freshwater and saltmarsh mosquitoes such as *Ae*. *vigilax* [106]. Gully traps, water-holes, drains, sewers and swamps, were also treated with DDT [108, 109] and there is a record for the use of DDT on *Culex fatigans* (*Cx*. *quinquefasciatus*) in 1947 which may indicate its use in drains and sewers [110]. By 1950, truck mounted thermal fogging machines were used to treat a range of insects including lice, cockroaches and flies [109]. The use of DDT in sewers, garbage tips and commercial premises may have contaminated many cryptic and subterranean sites. Although underground septic tanks were identified as habitat for other mosquito species, health officials ensured they were sealed from mosquitoes [42] and by 1975 the majority of Brisbane had a domestic sewerage system [111]. By the mid-1950s, resistance to DDT and the negative environmental effects were becoming apparent. The final mention of DDT use in council records was in 1954 [112] when it was replaced by pyrethrum and malathion [113]. There are no government records of DDT being used in residential buildings and no suggestion that DDT was commonly used indoors (where *Ae*. *aegypti* commonly rests [25]). It was unlikely used in rainwater tanks as authorities were aware of its negative health effects [114]. There is therefore no evidence that DDT was part of a successful large scale peri-focal spray strategy (like those used so successfully in the Americas).

#### The influence of climate

The role that climate plays on the distribution of *Ae*. *aegypti* in Australia is a complex subject and it remains unclear how long term weather patterns influenced the elimination from Brisbane. The city sits just outside the southern range of the current distribution of *Ae*. *aegypti* and authors have suggested that the climate is no longer suitable for the persistence of the species [63,17]. The relative suitability of the region remains debatable as there are more complex factors (such as the type and frequency of larval habitat in domestic environments) which influence the distribution of *Ae*. *aegypti* [115, 116]. In any case, Brisbane represents marginal habitat for the species when compared to some other cities in northern Australia, such as Cairns. We conclude, therefore, that the elimination process was enhanced by the suboptimal nature of the habitat and human agency.

### Conclusion

The elimination of *Ae*. *aegypti* from Brisbane incorporated elements of a top-down approach. Unlike the Soper campaigns in South America which used large scale residual chemical spraying to target larval habitat, the strategy in Brisbane was to intensively survey residential areas, and target larval habitat by insisting on screening rainwater tanks and removing all water-holding rubbish [43]. During the decade leading up to 1964, the majority of dwellings within Brisbane were surveyed for compliance with anti-mosquito regulation and, if in breach, were served with notices to rectify the situation. This top-down intervention identified key mosquito production hotspots and enforced compliance among all members of the population. These measures may be effective in small towns in arid and semi-arid areas where rainfall is limited [27]. However, with the modern proliferation of plastic containers and cryptic subterranean sites (like underground street drainage), the impact we have documented here may remain an artefact of history.

It is important that we continue to value the health initiatives that have safeguarded our cities in the past. The methodologies developed in Brisbane over many years show how a successfully integrated regulatory framework, with appropriate public engagement and enforcement can be applied to mosquito control. These historical records of management processes are rare, and this example shows how *Ae*. *aegypti* may be dramatically impacted at the margins of its range if key larval habitats are managed appropriately and enforced by effective legislation.

Drought conditions during the early 2000s have resulted in the installation of over 300,000 rainwater tanks in Queensland (>41% of properties in Brisbane). This has been driven by a culture of water harvesting via educational campaigns and legislated water restrictions [117]. Likewise, large numbers of water barrels and cisterns have been installed as a human response to drought throughout southern California and are likely to be an ongoing concern for local health authorities monitoring the spread invasive mosquito species [118]. As such, these regions should acknowledge the potential role of these behaviours on the reintroduction and spread of *Ae*. *aegypti*.

A clearer understanding of the threat posed by past epidemics and the enormous effort required to eliminate such a threat provides justification for ongoing surveillance by local and state authorities to ensure Australian cities remain vector free. With the current distribution limit of *Ae*. *aegypti* just north of Brisbane, ongoing mosquito surveillance and rainwater tank monitoring is essential. Although regulations are still in place, little is being done to inspect the condition of rainwater tanks, enforce anti-mosquito regulations, or educate residents on proper tank management within the Brisbane council area. The emergence and re-emergence of arboviruses including dengue, chikungunya and Zika viruses across the globe, further highlights the importance of early detection and response to invasive urban vectors. The successful campaign that led to the elimination of *Ae*. *aegypti* from Brisbane offers insights into the challenges we may face in the future, and provides an important source of knowledge from which to plan for and combat invasions of disease vectors internationally.

## Supporting information

S1 List Reference to historical survey locations not included in the dataset provided by [17]. (XLSX)

S2 List List of *Aedes aegypti* records from Brisbane that were used for analysis. (XLSX)

S1 Table References used in calculations of total house inspections, re-inspections, tanks surveyed, non-compliant tanks, tanks with mosquitoes present, notices and complied notices. (XLSX)

S2 Table Tank survey data used in calculations. (XLSX)
